# Catabolic flexibility of mammalian-associated lactobacilli

**DOI:** 10.1186/1475-2859-12-48

**Published:** 2013-05-16

**Authors:** Michelle M O’Donnell, Paul W O’Toole, Reynolds Paul Ross

**Affiliations:** 1 Teagasc Food Research Centre, Moorepark, Co. Cork, Fermoy, Ireland; 2 Microbiology Department, University College Cork, College Road, Co. Cork, Ireland

## Abstract

Metabolic flexibility may be generally defined as “the capacity for the organism to adapt fuel oxidation to fuel availability”. The metabolic diversification strategies used by individual bacteria vary greatly from the use of novel or acquired enzymes to the use of plasmid-localised genes and transporters. In this review, we describe the ability of lactobacilli to utilise a variety of carbon sources from their current or new environments in order to grow and survive. The genus *Lactobacillus* now includes more than 150 species, many with adaptive capabilities, broad metabolic capacity and species/strain variance. They are therefore, an informative example of a cell factory capable of adapting to new niches with differing nutritional landscapes. Indeed, lactobacilli naturally colonise and grow in a wide variety of environmental niches which include the roots and foliage of plants, silage, various fermented foods and beverages, the human vagina and the mammalian gastrointestinal tract (GIT; including the mouth, stomach, small intestine and large intestine). Here we primarily describe the metabolic flexibility of some lactobacilli isolated from the mammalian gastrointestinal tract, and we also describe some of the food-associated species with a proven ability to adapt to the GIT. As examples this review concentrates on the following species - *Lb. plantarum*, *Lb. acidophilus*, *Lb. ruminis*, *Lb. salivarius*, *Lb. reuteri* and *Lb. sakei*, to highlight the diversity and inter-relationships between the catabolic nature of species within the genus.

## Introduction

The human gut is an ecological niche where bio-transformations of dietary ingredients occur, catalysed by gut bacteria including lactobacilli. With that in mind, this review describes, compares and summarises the catabolic machinery present in the mammalian-associated lactobacilli. Lactobacilli are well-characterised members of the Lactic Acid Bacteria (LAB) that are found throughout the gastrointestinal tract of humans and other mammals, and although generally sub dominant in the colon, can be present at proportionately high levels in the upper GIT [1].

The LAB are low G+C Gram positive bacteria and have multiple uses in the food industry. Those associated with foods include the *Lactobacillus* and *Bifidobacterium* genera [2]. Bifidobacteria are phylogenetically distant from all of the other low [G+C%]–genome LAB, but are pragmatically included in the LAB group based on their functionality and habitat [3]. In this respect, LAB are integral inhabitants of the microbiota of the gastrointestinal tract where they contribute to intestinal barrier integrity and have roles in immunomodulation and pathogen resistance [4]. This adds impetus to their inclusion in functional food products.

The growth of all living organisms is dependent on efficient cycling and recovery of energy from the environment. Carbohydrates are the primary source of carbon and energy for the growth of microorganisms [5]. Glycolysis is the most important carbohydrate metabolic cycle in the majority of bacteria and constitutes the main energy generating mechanism. In many of the commensal *Lactobacillus* species, four of the main glycolytic genes along with a regulator are encoded by the *gap* operon. Such *gap* operons have previously been reported for other Gram positive bacteria including bacilli and clostridia [6,7]. The *gap* operon in mammalian lactobacilli generally encodes the central glycolytic gene regulator (*cgg*R), glyceraldehydes-3-phosphate dehydrogenase (*gap*), phosphoglycerate kinase (*pgk*), triosephosphate isomerase (*tpi*) and an enolase (*eno*). This operon arrangement was first noted in the genomes of *Lactobacillus plantarum* NC8 and *Lactobacillus sakei* Lb790 [8]. However, this particular arrangement of the *gap* operon has also since been identified in a variety of other *Lactobacillus* species genomes [9-13], while some other genomes contain only partial operons [14-18]. The conservation of this operon arrangement (and fragments thereof) in the genomes of a number of mammalian-associated lactobacilli has a number of implications. It suggests that, through evolution and adaptation, this glycolytic operon gene arrangement has been optimised for functionality and that there is a strong selective pressure against nucleotide, gene and operon change.

The ability of lactobacilli to efficiently utilise both of the glycolytic pathways facilitates the degradation of a wider range of carbohydrates present in a given niche, but is also information relevant for their industrial exploitation. For example, *Lactobacillus reuteri* is a commensal, facultatively hetero-fermentative species able to use both the Embden-Meyerhof pathway (EMP) and the phosphoketolase pathway (PKP) to ferment carbohydrates, exemplified by *Lb. reuteri* ATCC 55730 [19]. However, examination of the genome sequences of other heterofermentative lactobacilli has also revealed genes corresponding to both glycolytic pathways [10,14]. A number of genes for enzymes involved in both glycolytic cycles were identified in the genome of *Lb. reuteri* ATCC 55730; however, no recognisable *Lactobacillus*-like *pfkA* gene could be annotated. Metabolic flux analysis identified PKP as the main glycolytic pathway with EMP acting as a shunt [19]. Of the two glycolytic pathways, PKP yields less energy production overall. However, it seems that the EMP functions to provide a net gain in ATP in conjunction with the main energy production by the PKP. It is believed that the use of PKP as the main glycolytic pathway is an adaptation of *Lb. reuteri* and other heterofermentative lactobacilli to an environment rich in carbohydrates [19]. Since *Lb. reuteri* can be used as a cell factory to produce industrially exploitable metabolic intermediates or end products such as 3-hydroxypropionaldehyde for nylons and plastics, the ability to culture lactobacilli such as *Lb. reuteri* efficiently and cost-effectively will undoubtedly be informed by knowledge of its metabolism [20].

The structure of carbohydrates and their degrees of polymerisation determine the complexity of the sugar as well as the enzymes capable of degrading them. The building blocks of the majority of complex carbohydrates metabolised by LAB are glucose, fructose, xylose and galactose, while the linkages between monosaccharide residues are what determine carbohydrate digestibility in the small intestine [21]. Related to these parameters, prebiotics are defined as “selectively fermented ingredients that allow specific changes both in the composition and/or activity in the gastrointestinal microbiota that confers benefits upon host well-being and health” [22]. The lactobacilli of the mammalian microbiota are capable of fermenting a range of carbohydrates including oligosaccharides, starch, non-starch polysaccharides and many more carbohydrates [23-26]. Many different bacterial enzymes are used in the degradation of simple and complex carbohydrates; prominent among them are the glycosyl hydrolase (EC 3.2.1) family of enzymes [27,28]. Table 1 shows a list of glycosyl hydrolases commonly identified in and utilised by lactobacilli.

**Table 1:** Common glycosyl hydrolases present in mammalian lactobacilli

**Enzyme**	**EC number**	**Gene**	**Reaction**	**Associated pathways**	**References**
Alpha-amylase	3.2.1.1	amyA	Endo-hydrolysis of (1->4)-alpha-D-glucosidic linkages in polysaccharides containing three or more (1->4)-alpha-linked D-glucose units	Starch and sucrose metabolism	[9,10,14,26]
Oligo-1,6-glucosidase	3.2.1.10	malL	Hydrolysis of (1->6)-alpha-D-glucosidic linkages in some oligosaccharides produced from starch and glycogen by EC 3.2.1.1 (alpha-amylase), and in isomaltose	Starch and sucrose metabolism	[9-11,13,14,26]
Maltose-6’-phosphate glucosidase	3.2.1.122	glvA	Hydrolysis of maltose 6’-phosphate	Starch and sucrose metabolism	[18]
Alpha-glucosidase	3.2.1.20	malZ	Hydrolysis of terminal, non-reducing (1->4)-linked alpha-D-glucose residues with release of D-glucose	Galactose, starch and sucrose metabolism	[9-11,13,18,26,46]
Beta-glucosidase	3.2.1.21	bglX	Hydrolysis of terminal, non-reducing beta-D-glucosyl residues with release of beta-D-glucose	Starch and sucrose metabolism	[9-11,13,18,26]
Alpha-galactosidase	3.2.1.22	rafA	Hydrolysis of terminal, non-reducing alpha-D-galactose residues in alpha-D-galactosides, including galactose oligosaccharides, galactomannans and galactolipids	Galactose metabolism	[9-11,13,14,18,26,46]
Beta-galactosidase	3.2.1.23	lacZ	Hydrolysis of terminal non-reducing beta-D-galactose residues in beta-D-galactosides	Galactose metabolism	[9-11,14,18,26,46]
Beta-fructofuranosidase	3.2.1.26	sacA	Hydrolysis of terminal non-reducing beta-D-fructofuranoside residues in beta-D-fructofuranosides	Galactose, starch and sucrose metabolism	[9-11,13,14,18,26]
Beta-N-acetylhexosaminidase	3.2.1.52	nagZ	Hydrolysis of terminal non-reducing N-acetyl-D-hexosamine residues in N-acetyl-beta-D-hexosaminides	Amino sugar and nucleotide sugar metabolism	[9,10,14,26]
6-phospho-beta-galactosidase	3.2.1.85	lacG	Hydrolysis of 6-phospho-beta-D-galactosides	Galactose metabolism	[10,13,18]
6-phospho-beta-glucosidase	3.2.1.86	bglA	Hydrolysis of 6-phospho-beta-D-glucosyl-(1->4)-D-glucose	Glycolysis	[9-11,13,18,26]
Trehalose-6-phosphate hydrolase	3.2.1.93	treC	Hydrolysis of alpha,alpha-trehalose 6-phosphate	Starch and sucrose metabolism	[10,11,13,14,18]

In a more health conscious society, there has been a growing interest in recent years in the use of prebiotics as modulators of intestinal health [22], and prebiotics have become economically and industrially important as nutritional supplements for adults and as components in the burgeoning infant milk formula market. Lactose, soy oligosaccharides (stachyose and raffinose), lactulose and fructooligosaccharides are some of the carbohydrates that can be classed as prebiotics and that are commonly consumed as dairy, fruits and vegetables [29]. The microbiota is under constant pressure to adapt to the variety of foods consumed on a daily basis, especially in omnivores like humans. Lactobacilli present in the mammalian GIT have developed an array of adaptations to facilitate their continued presence in the human intestinal microbiota, examples of which will now be discussed. These case studies illustrate how knowledge of *Lactobacillus* metabolism is useful for optimizing their growth in the laboratory or factory, or promoting their retention in the intestinal tract by functional foods.

### Carbon metabolic machinery encoded by *lactobacillus* genomes and COG assignments

In the last decade, there has been a dramatic expansion in the number of available *Lactobacillus* genome sequences from organisms isolated from a variety of environments including the mammalian GIT, dairy products and fermented foods. Based on the Integrated Microbial Genomes (IMG) website (http://img.jgi.doe.gov/cgi-bin/w/main.cgi), as of April 2013 there are 46 completed *Lactobacillus* genome sequences, comprising 18 unique species. This expansion in the number of genome sequences available has facilitated the use of comparative genomic approaches to examine the machinery involved in growth and survival of lactobacilli with unprecedented rigour.

The genome size of a *Lactobacillus* is often a determinant of the organism’s capacity to metabolise a wide range of carbohydrates. Bacterial species with larger genomes are often capable of utilising a wider range of complex carbohydrates like prebiotics while those with smaller genomes are often associated with more restricted niche habitats, for example milk, and are only capable of utilising simple sugars like lactose and galactose. A comparison of the genome size and gene content for the majority of mammalian lactobacilli is shown in Table 2. *Lb. plantarum* WCFS1 has the largest genome of any *Lactobacillus* genome sequenced to date. This organism uses the phosphoketolase pathway as a central metabolic pathway. *Lb. plantarum* has been isolated from a variety of environments including soil, vegetables, meat, dairy and from the gastrointestinal tract of humans and animals and has been used as a model *Lactobacillus* for metabolic studies [30,31]. Indeed, the genome of *Lb. plantarum* encodes a large contingent of PTS transporters, ABC transporters and glycosyl hydrolases associated with carbohydrate metabolic flexibility [10]. In contrast, *Lactobacillus gasseri* has a much smaller genome and is considered to be part of the autochthonous species present in the human gastrointestinal tract, frequently isolated from the mouth, intestines, faeces and vagina of juveniles and adults [13,32]. This homofermentative organism is unable to ferment polyols (sugar alcohols), pentoses or deoxysugars, and in this respect resembles other obligate homofermenters [33,34]. Its inability to ferment pentoses is because of the absence of two key enzymes of the pentose phosphate pathway namely transketolase and transaldolase. Absence of either or both of these enzymes results in the inability to utilise pentose sugars. This limitation is also clearly illustrated by two members of the *Lb. salivarius* clade; *Lb. salivarius* itself (heterofermentative) produces both enzymes and is capable of utilising pentoses while *Lactobacillus ruminis* (homofermentative) lacks a transaldolase gene in its genome and as a result is unable to utilise pentose sugars [14,26].

**Table 2:** **Genome statistics of various mammalian *Lactobacillus *species**

**Genome name**	**Reference**	**Genome size (Mb)**	**Gene count**	**GC (%)**
*Lb. acidophilus *NCFM	[18]	1.99	1970	35
*Lb. amylovorus *GRL 1118	[16]	2.07	2126	38
*Lb. fermentum *CECT 5716	[35]	2.1	1125	51
*Lb. gasseri *ATCC 33323	[13]	1.9	1874	35
*Lb. johnsonii *FI9785	[36]	1.8	1804	34
*Lb. johnsonii *NCC 533	[11]	1.99	1941	35
*Lb. plantarum *JDM1	[37]	3.2	3026	45
*Lb. plantarum *WCFS1	[10]	3.35	3230	44
*Lb. reuteri *F275, JCM 1112	[17]	2.04	1901	39
*Lb. rhamnosus *GG	[12]	3.01	3016	47
*Lb. rhamnosus *GG, ATCC 53103	[38]	3.00	2905	47
*Lb. rhamnosus *Lc 705	[12]	3.03	3068	47
*Lb. ruminis *ATCC 25644	[9]	2.14	1901	44
*Lb. ruminis *ATCC 27782	[9]	2.01	2251	44
*Lb. salivarius *CECT 5713	[15]	2.13	1672	33
*Lb. salivarius *UCC118	[14]	2.13	2196	33

It should be emphasized, however, that examination of *Lactobacillus* genomes alone provides a limited quality of information. Functional genomics studies provide empirical experimental evidence for the functionality, mechanisms and pathways involved in carbohydrate metabolism. The fields of proteomics and transcriptomics in combination with genomics have been exploited to elucidate the mechanisms involved in carbohydrate metabolism in the host and this will be discussed in the next section.

### Metabolic potential of lactobacilli – adaptation to the environment

A wide range of adaptations can potentially develop within a genus or species based on the availability of nutrients and the complexity and competition within their current environment. Adaptation to a particular environment is of great importance for survival especially in a diverse and complex milieu like the mammalian gastrointestinal tract where a wide variety of carbon sources are often present.

*Lb. reuteri* has previously been used as a model organism for developing and testing microbe/host symbiosis theories [39]. Along with other mammalian associated lactobacilli, *Lb. reuteri* is reliant on the fermentable carbohydrates and amino acids present in the mammalian gut digesta. However, some strains of *Lb. reuteri* also have the ability to degrade 1,2-propanediol using the cobamide-enzyme-requiring propanediol dehydratase (EC 4.2.1.28), which may constitute a primary human colonisation parameter for the species. Propanediol dehydratase is a multifunctional enzyme with roles in glycerol utilisation, glycerolipid metabolism, vitamin B_12_ biosynthesis and reuterin formation [39]. Interestingly, an enzyme with a potentially similar function has been previously identified in *Lactobacillus brevis* ATCC 367 [40]. Glycerol is used in food and beverage manufacture as a sweetener, humectant, preservative, filler, thickening agent and solvent. It has also applications in the manufacture of mono/di-glycerides and poly-glycerol for margarine production. Therefore, glycerol can form a significant part of the foods consumed daily, particularly in the western world. The capability to hydrolyse glycerol may provide lactobacilli a competitive advantage in the gastrointestinal tract.

Some *Lactobacillus* species utilise differentially present or differentially expressed features of their carbohydrate metabolic machinery in order to facilitate their colonisation and persistence in the mammalian gut. For example, *Lactobacillus johnsonii* and *Lb. reuteri* do not compete in the mouse fore-stomach because the former utilizes glucose and the latter maltose, even though both species have the genes for metabolizing both substrates [41]. This is an example of niche sharing by way of resource partitioning. Using a mouse model Denou *et al*., 2008 showed that *Lb. johnsonii* strains use a number of genes (carbohydrate utilisation genes included) for long-term gut persistence. Correlating the datasets from the genomic hybridisation of two strains (ATCC 33200 and NCC533) and the *in vivo* microarray transcription data from strain NCC533 identified six genes, forming three loci that are *Lb. johnsonii* NCC533 strain specific. Two of the loci are involved in carbohydrate metabolism namely exo-polysaccharide biosynthesis (glycosyltransferases) and a mannose phosphoenolpyruvate phosphotransferase system PTS (transporter) [42].

A similar transcriptomic study, focusing on the adaptations of *Lb. plantarum,* demonstrated the capacity of a *Lactobacillus* to alter its metabolism in response to the human or murine intestine [43,44]. In those studies, a number of genes required for carbohydrate metabolism were identified as differentially transcribed in the human and mouse gastrointestinal tract under different dietary conditions. The genes up-regulated included those encoding glycosyl hydrolases, glycolytic enzymes and various carbohydrate transporter classes [43,44]. An overlap in the enzymes induced in the mammalian GIT included those involved in the degradation and transport of lactose and the plant derived-disaccharides melibiose, cellobiose and maltose. In animals fed a Western diet there was also a noteworthy up-regulation of glycerol metabolism-related enzymes, which relates to the presence of glycerol in many foods discussed above. The induction of carbohydrate metabolism genes highlights the importance of metabolic flexibility in the adaptation of *Lactobacillus* and other bacteria to the human and mammalian intestine [43,44].

Metabonomic studies using Nuclear Magnetic Resonance (NMR) spectroscopy have identified the metabolites most affected by supplementation of the human diet with fructooligosaccharides (FOS) and *Lactobacillus acidophilus* and *Bifidobacterium longum* based synbiotics [45]. Beneficial short chain fatty acids (SCFA) namely propionate and butyrate were identified in faeces of individuals receiving the synbiotic treatments. There was also a marked decrease in the recoverable amino acids in the samples. The increase in *lactobacillus* numbers over the month-long period as well as the increase in SCFA levels and decrease in amino acid concentrations indicate that the feeding of a synbiotic resulted in a shift of the intestinal metabolome from an overall proteolytic pattern to a saccharolytic one. The presence of FOS in the diet, which is indigestible in the upper GIT, had the ability to affect the SCFA profile of the lower GIT when fermented by bacterial species like lactobacilli and bifidobacteria [45].

Another recent study focussed on the adaptation by *Lb. reuteri* to the GIT of mice [46]. *In vivo* studies using *Lactobacillus*-free (LF) mice and different vertebrate-derived *Lb. reuteri* isolates established that only the rodent isolates were capable of reaching colonising numbers in the LF mice, supporting the theory of host specialisation. Using comparative genome hybridisation, the genome of an *Lb. reuteri* mouse isolate was compared to that of 24 other *Lb. reuteri* strains from various sources. A xylose utilisation operon was conserved in the strains of rodent and porcine origin [46] but absent in the others. Xylose forms a large percentage of the hemi-cellulose found in some plants and so is consumed as part of animal diet.

Other examples of niche-specific genes or host specialisation genes between dairy and gastrointestinal lactobacilli have also been revealed using comparative genomic approaches. For example, mannose-6-phosphate glucosidase (EC 3.2.1.122), a mannose catabolic enzyme, was identified as a solely gut-specific gene in the genome sequences of a number of frequently present mammalian lactobacilli [11,12,16,18,37,47]. This enzyme works in conjunction with a maltose phosphotransferase system to import phosphorylated maltose into the cell. Once internalised the enzyme converts maltose-6-phosphate into glucose and glucose-6-phosphate, and it is this method of transport and degradation that is thought to be specific to strains of gut origin. However, this mechanism of maltose utilisation is not ubiquitous among the gut lactobacilli [9,10,13,14,47]. Genome decay, due to gene loss, seems to operate in the dairy lactobacilli that have higher numbers of pseudogenes in their genomes than other lactobacilli. The majority of the pseudo-genes present are related to carbon catabolism, amino acid metabolism and transport, reflecting the fact that these organisms (for example *Lactobacillus helveticus*[48]) have less need for these processes in a milk environment. However, it must be noted that even for an organism like *Lb. plantarum* with a diverse range of habitats, continual passage in a nutrient rich medium can lead to genome contraction and loss of certain types of carbohydrate transporters and enzymes [37]. A genome level comparison of *Lb. plantarum* JDM1 with *Lb. plantarum* WCFS1 revealed that certain saccharolytic genes and transporters present in strain WCFS1 were absent in the closely related strain JDM1 [10,37]. Examples of the absent enzymes include alpha-amylase, alpha-L-rhamnosidase, beta-N-acetylhexosaminidase, mannosyl-glycoprotein, endo-beta-N-acetylglucosaminidase and glucan 1,4-alpha-maltohydrolase [37]. This variability of saccharolytic capability within a species is also clearly illustrated by the work of Molenaar *et al*., 2005 who compared over 20 *Lb. plantarum* species using microarray genotyping technology [49]. These were clear examples of a species adapting to their environment and altering their metabolic profile to suit the new environment either by gene acquisition or in this case gene loss.

Recent studies have also focussed on the cellular response of certain lactobacilli to complex carbohydrates. For example, Majumder and colleagues identified a number of proteins involved in the adaptation of *Lactobacillus acidophilus* NCFM to growth in the presence of the prebiotic lactitol (a synthetic sugar alcohol derived from lactose, used in the food industry and in some medications) [50]. Examination of the late exponential phase whole-cell extract proteome revealed a number of proteins present which may be involved in utilization of lactitol including a β-galactosidase subunit, galactokinase and other galactose utilisation proteins. The majority of enzymes identified in lactitol utilisation were the same enzymes involved in the Leloir pathway (the lactose utilisation pathway) and transportation of lactitol into the cell was facilitated by LacS (a glycoside-pentoside-hexuronide cation symporter). While transport of lactitol is facilitated by a permease, it is the phosphotransferase system that transports and metabolises sorbitol [50]. *Lb. reuteri* (as well as the other mammalian lactobacilli) also possess the genetic determinants for enzymes associated with the utilisation of raffinose family oligosaccharides (RFO). RFOs are present in many vegetables namely legumes and are associated with flatulence and gastrointestinal upset [51]. Alpha galactosidase (EC 3.2.1.20) and to a lesser extent levansucrase (EC 2.4.1.10) are the main enzymes commonly encoded in the genome sequences of mammalian derived lactobacilli, which are responsible for the hydrolysis and partial hydrolysis of RFO, respectively [10-14,16,18,26,35,52]. Interestingly, the genome sequences of dairy lactobacilli such as *Lactobacillus bulgaricus* and *Lb. helveticus*[48,53] are devoid of RFO degradation associated enzymes, consistent with the fact that milk generally contains negligible amounts of RFO.

Dairy derived lactobacilli, however, can possess considerable and demonstrable metabolic flexibility. Burns *et al*., 2010 investigated the “progressive adaptation” of dairy *Lactobacillus delbrueckii* strains to bile (a bio-surfactant produced in the liver for emulsifying fats in the diet). The proteomes of *Lb. delbrueckii* and an enhanced bile resistant derivative were examined using cells grown in the presence and absence of bile. A total of 35 proteins were affected by the inclusion of bile. Three of the proteins were found to be part of the glycolytic cycle with phosphoglycerate mutase (*pgm*) and glyceraldehyde-3P-dehydrogenase genes up-regulated, while fructose-bisphosphate aldolase was down-regulated at the protein level [54]. *Lactobacillus casei,* a predominantly dairy associated isolate, is frequently isolated from a range of other niches, including plants, and the human GIT [55,56]. Examination of the *Lb. casei* strain fermentation profiles from these various niches identified several trends, for example the increased utilisation of polyols by strains of plant and human origin. Not surprisingly, strains of cheese origin also were found to have an increased capacity for lactose utilisation when compared to non-dairy isolates. The data suggest that *Lb. casei* can adjust its metabolic capabilities in order to adapt to the carbon sources available in a particular niche.

Lactobacilli also have the capacity to alter their metabolism to adapt to a new environment. This is clearly exemplified by a study of *Lb. sakei* where Chiaramonte and colleagues (2010) showed that the meat-borne *Lactobacillus sakei* is capable of colonizing the GIT of mice [57]. Analysis of *Lb. sakei* wild-type and morphological mutants revealed an increased capacity for the utilisation of some carbon sources (fructose, ribose and galactose) when compared to the original meat-borne parent strain. Up-regulation of the genes encoding 6-phosphofructokinase, L-lactate dehydrogenase and fructose-bisphosphate aldolase was considered to be the likely cause of this capacity to colonize the mouse GIT. Two genes involved in nucleotide metabolism, CTP synthase and xanthine phosphoribosyltransferase were also up-regulated in the mutants derived from the passage of meat-borne *Lb. sakei* strain through the GIT of axenic mice [57].

### Transporters and their importance in metabolic flexibility and regulation of metabolism

Carbohydrate transporters or permeases are an essential component in carbohydrate metabolism to facilitate permeability of the cell to carbon metabolites, and may be the rate limiting step in their utilization [58]. Transporters involved in carbohydrate metabolism include proton coupled active transport and group translocators [59]. A summary of those systems most commonly found in lactobacilli is presented in Table 3.

**Table 3:** Common carbohydrate transporters utilised by mammalian lactobacilli

**Superfamily**	**Transport family**	**Transporter class**	**Transporter subclass**	**Transport classification system**	**Transmembrane domain range**
MFS	Major Facilitator Superfamily (MFS)	Electrochemical Potential-driven Transporters	Porters (uniporters, symporters, antiporters)	TC 2.A.1	12-24
GPH	Glycoside-Pentoside-Hexuronide (GPH):Cation Symporter Family	Electrochemical Potential-driven Transporters	Porters (uniporters, symporters, antiporters)	TC 2.A.2	12
ATP Binding Cassette	ATP-binding Cassette (ABC)	Primary Active Transporters	P-P-bond-hydrolysis-driven transporters	TC 3.A.1	5-6
PTS-GFL	PTS Glucose-Glucoside (Glc) Family	Group Translocators	Phosphotransfer-driven Group Translocators	TC 4.A.1	8
PTS-GFL	PTS Fructose-Mannitol (Fru) Family	Group Translocators	Phosphotransfer-driven Group Translocators	TC 4.A.2	8
PTS-GFL	PTS Lactose-N,N’-Diacetylchitobiose-β-glucoside (Lac) Family	Group Translocators	Phosphotransfer-driven Group Translocators	TC 4.A.3	8
PTS-GFL	PTS Glucitol (Gut) Family	Group Translocators	Phosphotransfer-driven Group Translocators	TC 4.A.4	8
PTS-GFL	PTS Galactitol (Gat) Family	Group Translocators	Phosphotransfer-driven Group Translocators	TC 4.A.5	8
PTS-GFL	PTS Mannose-Fructose-Sorbose (Man) Family	Group Translocators	Phosphotransfer-driven Group Translocators	TC 4.A.6	8
PTS-GFL	PTS L-Ascorbate (L-Asc) Family	Group Translocators	Phosphotransfer-driven Group Translocators	TC 4.A.7	8

Within the LAB, the ATP binding cassette (ABC) transporters form the largest group [60], whereby a metabolite or macromolecule is transported using energy derived from ATP hydrolysis [61]. ABC transporters are capable of transporting mono, di, tri, poly and oligosaccharide as well as polyols [62]. ABC transporters encoded by the genome sequences of mammalian lactobacilli include those for maltose, lactose, arabinose, sorbitol, mannitol, glucose, N-acetylglucosamine and cellobiose transport together with ribose, xylose, fructose and rhamnose, all of which are commonly found in the mammalian digesta, especially of omnivores [9,10,13,14,18]. However, genomes from strains of dairy and meat origin so far examined harbour only gene fragments of carbon-transport-related ABC transporters and do not therefore encode a complete transporter protein [53,63].

Transporters that use chemo-osmosis in order to import carbohydrates are called secondary active transporters and are categorised as either uni-porters, symporters or anti-porters [64]. The majority of uni/sym/anti-porters are part of a large group called the Major Facilitator Superfamily (MFS) with over 40 recognised MFS families [65]. MFS transporters are capable of transporting the majority of micro-molecules (like low DP carbohydrates) but are unable to transport macromolecules. Glycoside-pentoside-hexuronide (GPH) transporters are a class of sodium ion symporters that are used by both homo and heterofermentative lactobacilli to transport carbohydrates [10,23,26,43,66]. Lactobacilli found exclusively in the gastrointestinal tract, for instance *Lb. ruminis,* have been found to harbour a lower number of complete PTS transporters but a higher number of symporters otherwise known as secondary active transporters [26]. In contrast, *Lb. gasseri,* another autochthonous species in the human gut, encodes two glucose permeases but does not encode a lactose/galactose permease [13]. The reliance of some lactobacilli on symporters may be due in part to the fact that the gastrointestinal tract is a nutrient-rich, complex environment. Thus the cells do not have to expend as much energy in order to internalize carbohydrates; instead a carbohydrate is transported into the cell using simultaneous sodium ion exchange. Often the sugars found in the GIT are of a high degree of polymerisation like inulin and starches which require alternate transportation methods to the PTS system.

The majority of carbohydrate transport in lactobacilli isolated from a variety of environments, for example *Lb. plantarum* and *Lb. acidophilus,* is done using PTS systems [10,18]. This method of transport involves the coupling of energy molecules with phosphorylation, to bring the phosphorylated carbohydrates into the cell, and is of particular importance in the transport of low complexity hexose sugars [67]. PTS transporters are characterised by a phosphate transfer cascade involving phosphoenolpyruvate (PEP), enzyme I (EI), histidine protein (HPr) and various EIIABC’s. HPr is phosphorylated at site serine 46 by HPrK/P which is only present in the low [G+C%] Gram positives [68]. PEP-dependent phosphorylation of HPr by EI yields HPr-His-P, which is required for PTS-mediated transport of carbon sources [69].

Many mammalian lactobacilli rely on the PEP-PTS to facilitate nutrient uptake in the gastrointestinal tract and contain a number of PTS classes. This is best exemplified by *Lb. plantarum* and members of the acidophilus complex [11,13,18]. The *Lb. plantarum* WCFS1 genome encodes 25 predicted complete PTS EII complexes; it also encodes some incomplete complexes [10]. This high number of PTS genes is one of the largest counts in a sequenced microbial genome and currently comes second only to *Listeria monocytogenes*[70]. The genome of *Lb. acidophilus* NCFM encodes 20 PEP-PTS; the transporters have predicted specificity for trehalose, fructose, sucrose, glucose, mannose, melibiose, gentiobiose, cellobiose, salicin, arbutin and N-acetylglucosamine PTS [18]. The genome of *Lb. gasseri* ATCC 33323, another acidophilus complex bacterium, encodes 21 PEP-PTS transporters including those for predicted transport of fructose, mannose, glucose, cellobiose, lactose, sucrose, trehalose, β-glucosides and N-acetylglucosamine [13]. The genome of *Lb. johnsonii* NCC 533 encodes 16 PEP-PTS which is a large number for a genome of its size; allowing the predicted transport of sugars such as mannose, melibiose, cellobiose, raffinose, N-acetylglucosamine, trehalose and sucrose ,which is supported experimentally by physiological (API CH50, Biomerieux, France) data [11].

As mentioned above, bacterial species will often preferentially utilise one carbohydrate prior to utilising another by means of the phosphotransferase system. This system requires strict regulation to ensure the ability to preferentially utilise the particular carbohydrate, for example glucose, before any other carbon source. This type of control is called carbon catabolite repression (CCR). CCR is defined as “a regulatory phenomenon by which the expression of functions for the use of secondary carbon sources and the activities of the corresponding enzymes are reduced in the presence of a preferred carbon source” [71]. Various methods of CCR are present in nearly all free living microorganisms. In phylum Firmicutes, the main components are catabolite control protein A (CcpA), HPr, HPr kinase/phosphorylase (HPrK) and the glycolytic enzymes fructose 1,6-bisphosphate and glucose-6-phosphate. In *Enterobacteriaceae* the phosphorylation state of EIIA is crucial for CCR, whereas in Firmicutes the phosphorylation state of HPr is essential [72]. HPr phosphorylation can occur at two sites, at Histidine-15 by EI and at Serine-46 by HPrK. In the presence of glucose, there is an increase in the level of fructose 1,6-bisphosphate which indicates a high level of glycolytic activity. HPrK kinase activity is triggered by this increase causing phosphorylated HPr to bind to CcpA, which then binds to the cre site on the DNA thereby repressing transcription of the catabolic genes. When glucose levels are low there is a decreased level of Fructose 1,6-bisphosphate, which dephosphorylates HPrK/P at Ser-46 [73,74]. The outcome from CCR is the same with the preferential use of a carbon source.

Regulation of carbohydrate metabolism (especially lactose) has also been identified in *Lb. acidophilus* NCFM [50]. In the presence of lactose there was an increase in the abundance of pyruvate kinase, a noted indicator of regulation via carbon catabolite repression, and the down regulation of genes for nucleotide metabolism proteins [50]. A similar phenomenon was noted in the proteome of *Lactococcus lactis* when grown in the presence of lactose as a carbon source [75]. Similarly, in *Lb. plantarum* CCR has been shown to control the expression of phospho-β-glucosidase [76]. Lactobacilli like *Lb. brevis* and *Lb. pentosus* which have relaxed control of their carbon catabolite machinery are being investigated for their carbon degradation potential for industry [77,78]. This alternative or relaxed mechanism of carbon catabolite control is being used in industrial fermentations of cellulolytic and ligno-celluloytic materials to form lactic acid and ethanol, respectively [77,78]. The use of lactobacilli that are capable of using mixed carbohydrate sources for growth is of great importance for industries utilising lignocellulose hydrolysate-like biomass containing hexose and pentose sugars like glucose, arabinose and xylose.

### Horizontal gene transfer and plasmid-encoded carbon metabolism genes

Horizontal gene transfer (HGT) has long been recognised as a method by which bacteria receive genes and other genetic elements conferring new abilities from another species, for example *Escherichia coli* transferring ampicillin resistance to *Shigella flexneri*[79]. Mobile genetic elements include transposons, bacteriophages and plasmids [80]. While examining the genomes of two species of GIT-associated lactobacilli and a dairy isolate in particular (*Lb. delbrueckii* ssp. *bulgaricus*), it was noted that extensive horizontal gene transfer (HGT) had occurred between the three species [81]. Comparison of phylogenetic trees for over four hundred proteins highlighted the variance between the members of the acidophilus complex. In many cases, the acquisition of new genetic capabilities can include a new method of solute transportation. Mannose PTS transporters are a class of PTS transporters (TC 4.A.6) associated with mammalian associated *Lactobacillus* species with the exception of *Lb. reuteri*[17]. Comparison of phylogenetic trees created from the ClustalW alignment of mannose PTS transporters from twenty five bacteria including *Lb. plantarum*, highlighted the likelihood of HGT having occurred [82]. The study identified the lack of concordance between evolutionary data from 16S ribosomal RNA gene sequences and the evolutionary data generated from the mannose PTS sequences. The analysis also noted that within the mannose transporters in particular, there was a high level of sequence variation among the bacteria studied. Sequence analysis and comparison of the 58 mannose PTS proteins identified the varying patterns caused by HGT and allowed organising the species into six groups [82].

A plasmid is defined as “a linear or circular double-stranded DNA that is capable of replicating independently of the chromosomal DNA”. Plasmids are very common within the *Lactobacillus* genus with approximately 38% of all species containing one or more plasmids of varying sizes [83], including most of the species routinely used for industrial applications. Regions of homology have been identified in plasmids from the same species, genus and from other genera [84]. Plasmids contribute to horizontal gene transfer, with plasmids often containing genes for carbohydrate, citrate and amino acid utilisation, production of bacteriocins or other biosynthetic genes [83]. This is best exemplified by *Lb. salivarius* UCC118 which contains 2 cryptic plasmids and one megaplasmid [85]. The megaplasmid (pMP118) harbours genes for the utilisation of pentoses and polyols. It also carries genes involved in glycolysis (FBP) and genes for two pentose pathway essential enzymes, transketolase and transaldolase. The plasmid pMP118 encodes an additional copy of the enzyme ribose-5-phosphate isomerase which may contribute to its metabolic flexibility and adaptive capabilities. Thus, for *Lb. salivarius* to survive in an environment dominated by pentose sugars these plasmid acquired genes would be essential [14,85]. However, the most striking example in the mammalian derived lactobacilli of the importance of plasmids in carbohydrate metabolism is the case of the *Lactobacillus rhamnosus* Lc705 plasmid pLC1 [12]*.* This 64 Kbp plasmid sequence encodes proteins predicted for the fructose PTS, glucose uptake proteins, a glycosyl hydrolase and a number of genes involved in alpha and beta-galactoside utilisation and transport [12]. It is obvious that without the presence of these plasmid-borne genes, *Lb. rhamnosus* Lc705 would be at a severe competitive disadvantage in the mammalian GIT compared to other *Lactobacillus* species that have these genes integrated in the chromosome. The presence of these genes in the plasmid presumably allows *Lb. rhamnosus* to compete for the alpha galactosides and fructose from plant sources and also for the beta-galactosides from dairy products. It is clear from the available plasmid sequences that, while not always present, carbohydrate genes carried by plasmids are important mobile genetic elements for lactobacilli.

The presence of carbohydrate metabolic genes located on plasmids is also common in food, plant and dairy lactobacilli. Another example of plasmid encoded pentose sugar utilisation genes is the xylose utilisation cluster present in plasmids isolated from *Lactobacillus pentosus*[86], a plant derived *Lactobacillus*. A study comparing 34 sequenced *Lactobacillus* plasmids revealed that the carbohydrate and amino acid transport category was that most frequently encoded among the plasmids analysed [87]. The presence of a larger cohort of carbohydrate and amino acid transporters is possibly a niche adaptation. *Lb. casei* 64H lacking the plasmid pLZ64, which contains a lactose PEP-PTS and phospho-β-galactosidase, is unable to utilize lactose. There is limited knowledge on the true extent of plasmids from mammalian derived lactobacilli and their impact on gut health. However, there is detailed knowledge on the presence and function of plasmids in dairy-derived lactobacilli for example *Lb. casei*[88].

## Conclusions

Carbon metabolism is essential for life and the survival of many bacterial species depends on their ability to exert some degree of metabolic flexibility. *Lactobacillu,s* as a genus, has a broad range of environmental niches and is equipped with an intricate array of enzymatic systems and adaptive responses to cope with differing carbohydrate sources. This poses challenges for examining the effect of lactobacilli on the gut microbiota but also opportunities for their efficient industrial exploitation. Although there is an extensive amount of information on the *in vitro* and *in silico* catabolic flexibility of mammalian lactobacilli, additional studies and investigations are required to elucidate all the factors and systems that are involved in carbohydrate degradation mechanisms *in vivo* in the mammalian GIT. Further metabolomic, metabonomic and metatranscriptomic studies along with concerted effort are needed to fully elucidate all of the effects that carbohydrate metabolism has on strain phenotypes. With advances in sequencing technologies it is now possible and “affordable” to use RNA-seq (whole transcriptome shotgun sequencing) rather than using microarrays. Microarrays have shortcomings that including for example requiring prior sequence information of a strain, and the need to use of pure cultures which makes it difficult to assess the effect of species or carbohydrate on the microbiome as a system of interconnected genera and species. Metatranscriptomics can identify the gene expression of mixed communities of organisms *in vivo* under a wide range of parameters including diet, stresses, disease state and other environmental and health factors. The use of metatranscriptomics in conjunction with animal model feeding studies would allow a more accurate measurement of the effect diet has on the *Lactobacillus* component of the microbiota. For *in vivo* studies the use of a “standard” mammalian GIT model, for example the pig, whose physiology is similar to that of humans would be advantageous in allowing more rigorous comparisons of *in vivo* feeding studies. The use of mouse models, while convenient and relatively inexpensive, should be viewed as a “small-scale” step before transitioning the research into a larger human GIT analogue model like the pig. Further investigations using some of the techniques outline above on a wider number of mammalian derived lactobacilli will provide information that will lead to a greater understanding of *in vivo* carbohydrate metabolism of mammalian derived lactobacilli and the implications for human and animal health. The industrial usage of lactobacilli for production of metabolites and process ingredients will benefit from progress in metabolic modelling, exemplified to date by *Lb. plantarum* WCFS1 [89], but not yet applied to many relevant lactobacillus species. Success of these modelling experiments will be aided by empirical data provided by complementary “omics” analyses, generating greater precision in establishing and fine-tuning models for lactobacillus growth in the laboratory and in the factory.

## Competing interest

The authors declare that they have no competing interests.

## Authors’ contributions

MMOD drafted the manuscript and participated in the title conception, PWOT and RPR conceived of the title, and helped to draft the manuscript. All authors read and approved the final manuscript.
